# Endovascular treatment of a traumatic thoracic pseudo-aneurysm in a pediatric patient: a case report with review of literature

**DOI:** 10.1186/s13019-023-02265-7

**Published:** 2023-05-18

**Authors:** Muniba Afzal, Safaa Abdulreda Najar, Hassan Baghazal, Noora Alshahwani

**Affiliations:** 1 General Surgery Department, Hamad Medical Corporation, Doha, Qatar; 2 General and Thoracic Surgery, Sidra Medicine, Doha, Qatar

**Keywords:** Aortic injury, Pseudo-aneurysm, TEVAR, Blunt thoracic injury

## Abstract

Blunt aortic injury (BAI) as a result of thoracic trauma is a rare entity in the adult and pediatric population. The endovascular approach has been the preferred method of management over operative repair in adults. However, data on pediatrics is limited to case reports and case series with no long-term follow-up. There are no current guidelines for management in the pediatric population. We are reporting a successful repair of a traumatic thoracic aortic aneurysm in a 13 year old boy with covered stents, with a review of relevant literature.

## Introduction

Traumatic vascular injury in general and thoracic aortic injury in specific is relatively rare in children and adolescents compared to the adult population [1]. The mechanism of injury in such cases is most often blunt rather than penetrating trauma. Heckman et al. reports an incidence of < 1% for blunt traumatic aortic injury (BTAI) in the pediatric population using the data from US National Trauma Database [2]. A slightly higher incidence in the range of 1.5–2% is reported in adults [3]. The presence of such injury reflects on the severity of the mechanism and the possibility of other associated life-threatening injuries. Data from the National Pediatric Trauma Registry reports an overall mortality of 15% for children with blunt thoracic injury. The pediatric population is anatomically predisposed to thoracic injury. One of the reasons is the increased compliance of the chest wall due to incomplete rib ossification and a cartilaginous chest wall [4]. In addition, because of a relatively small volume to body surface area, children are at higher risk for injuries to multiple organs after blunt trauma [5–7].

The current society of vascular surgery classification grades aortic injury as minimal aortic injury (MAI) and significant aortic injury (SAI) based on the absence or presence of external aortic wall deformity, respectively [8]. MAI and SAI are further divided into four types ranging from Type I which is Intimal tear or flap to type IV which is open rupture. While there have been multiple studies evaluating the efficacy and success of conservative management of MAI, most of the cases of SAI have to undergo either endovascular or operative repair [9]. Below is a case report of a successful repair of traumatic thoracic aortic aneurysm in a 13 year old boy with covered stents, and a review of the literature for similar cases.

## Case report

**Fig. 1:**
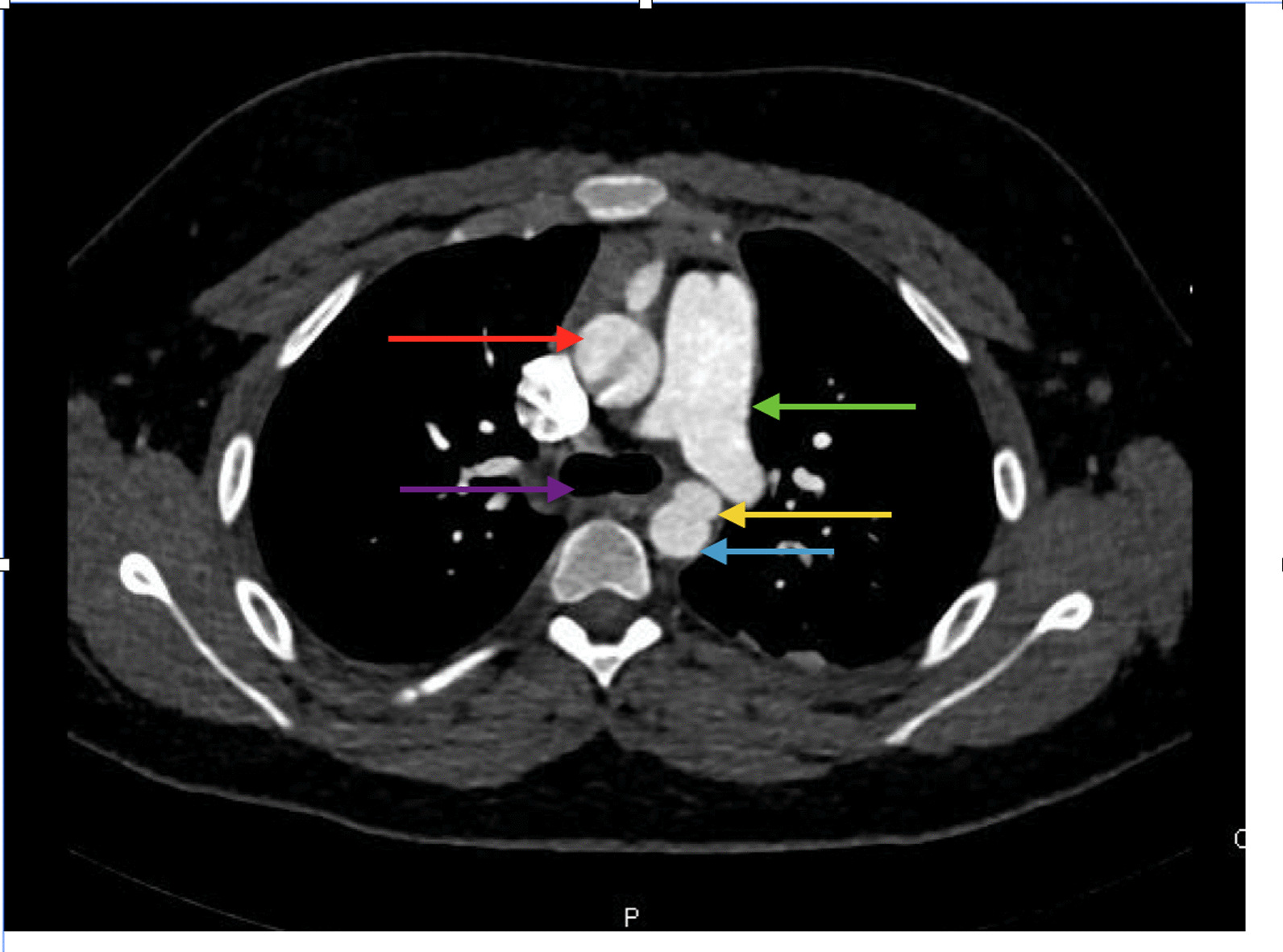
ECG-gated CT angiography showing the pseudoaneurysm at the distal aortic arch. Yellow arrow: pseudoaneurysm; red: ascending aorta; blue: descending aorta; green: pulmonary trunk; purple: tracheal bifurcation

A previously healthy 13-year-old boy was brought to hospital following a motor vehicle accident. He was a front-seat passenger and was unrestrained. Speed at the time of impact was not known. At the time of presentation, he was alert and oriented with a Glascow-Coma Scale (GCS) of 15. Examination revealed a deep scalp laceration on the right side, tenderness over the right chest, left pelvic brim and restricted movement of the right arm and left leg. On X-ray imaging, the patient had multiple fractures including the right clavicle, the lower third of the sternum, the second to seventh ribs on the right, and the right sacral ala. Computed tomography (CT) showed an additional pulmonary contusion and low grade hepatic and splenic lacerations. There was a focal bulge of the anterior aspect of the distal aortic arch, with a thin periaortic hematoma, suggestive of a traumatic pseudoaneurysm. For further evaluation of the pseudoaneurysm, the patient underwent ECG gated CT Angiography. It demonstrated a saccular bulge suggesting pseudoaneurysm at the level of the aortic isthmus, measuring 1.1 long × 1.4 × 0.8 cm. The diameter of the aorta proximal to the aneurysm was 13 mm.There was no extra-luminal leakage of contrast to suggest rupture (Fig. 1). The rest of the thoracic aorta appeared unremarkable.

The patient’s initial management included fluid resuscitation, pain management and blood pressure control along with conservative management of other injuries. He was transferred afterwards to the pediatric tertiary care center. A multi-disciplinary team discussion involving pediatric surgery, vascular and cardiac surgery led to the decision to defer any acute treatments to allow for the recovery from associated injuries, and to attempt endovascular stenting after ensuring hemodynamic stability and that there is no ongoing blood loss. During this period, the patient was treated conservatively with blood pressure control and analgesics.

**Fig. 2:**
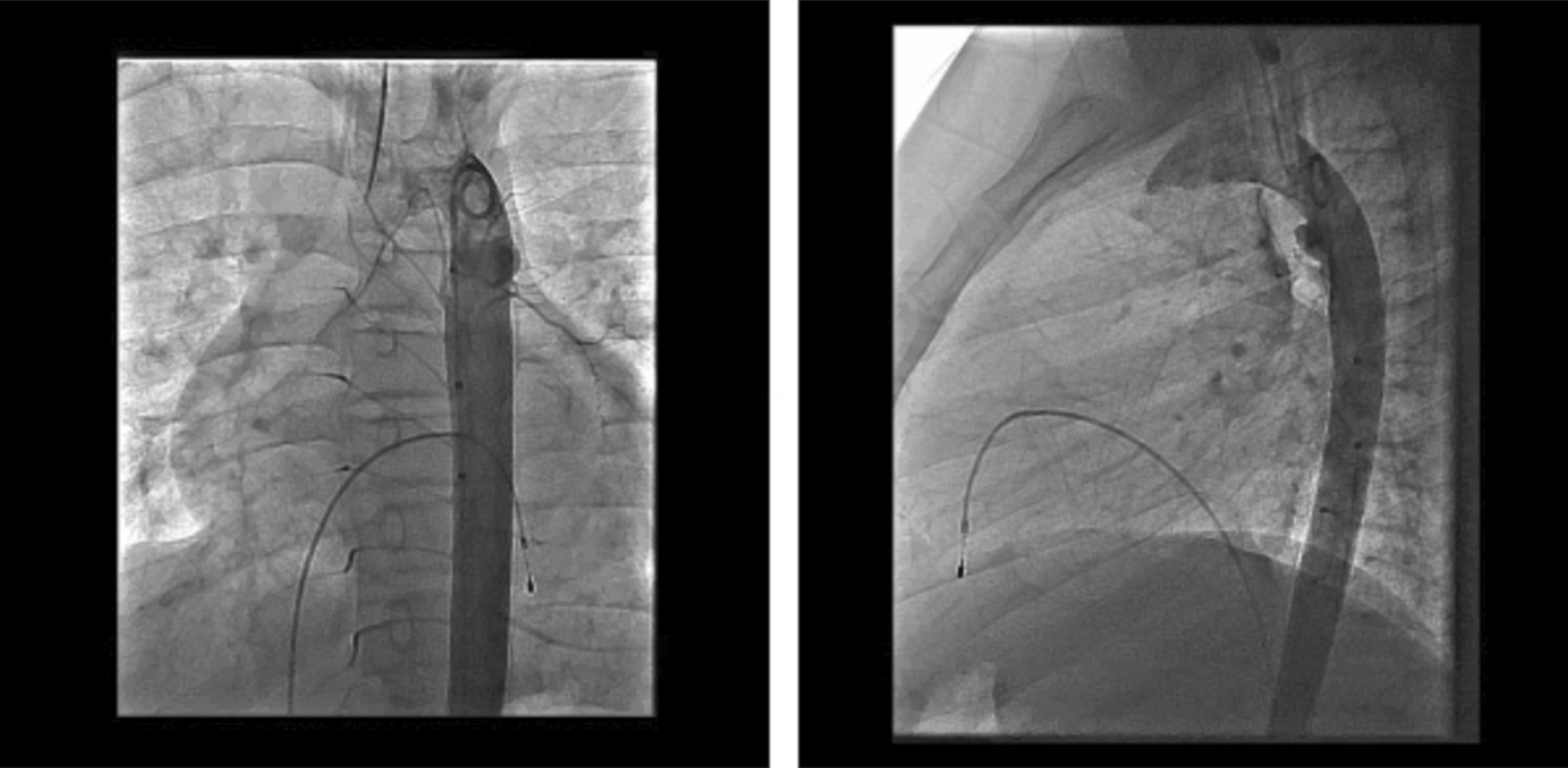
Baseline aortogram showing aneurysm

**Fig. 3:**
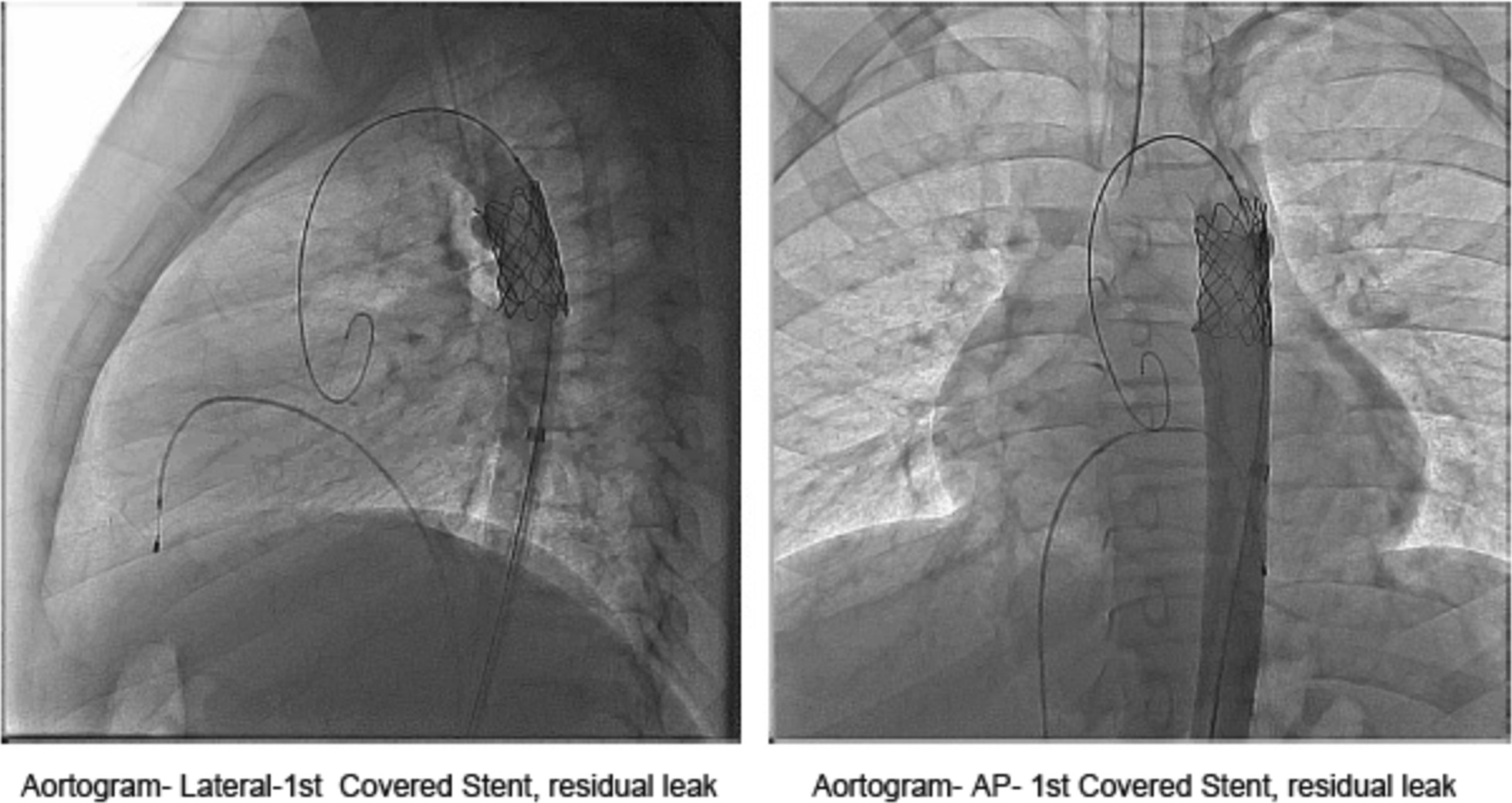
Aortogram showing residual leak after 1st stent placement

**Fig. 4:**
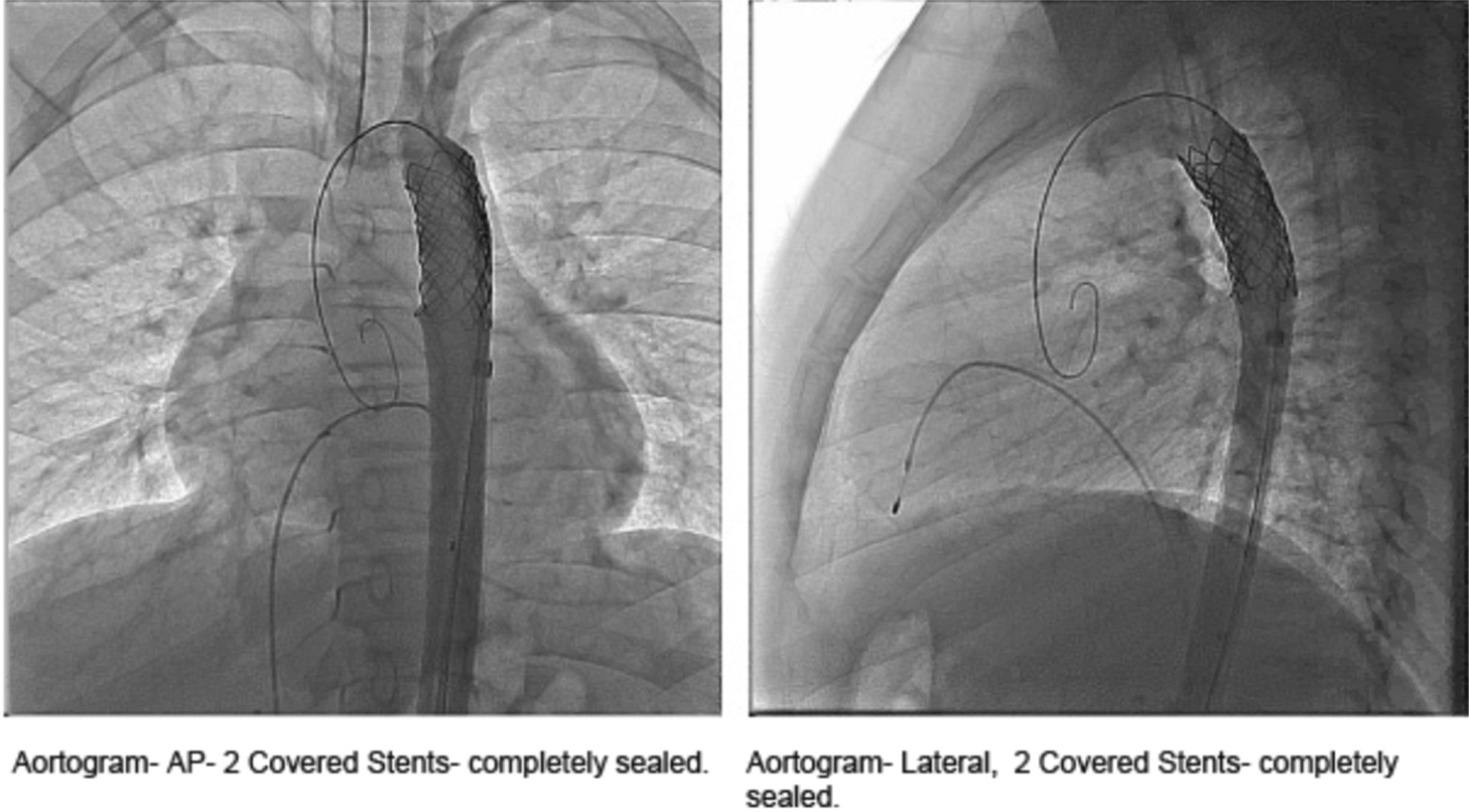
Aortogram with overlapping 2 stents completely covering the aneurysm

After a week of ICU care, the patient underwent endovascular aortic stenting. Access was obtained through the right femoral artery. Baseline aortogram showed the aneurysm of descending thoracic aorta distal to left subclavian artery (Fig. 2). A 39 mm long covered CP stent (cheathum platinum) from NuMED ® was placed at the site of the aneurysm using a 20 mm BiB balloon. An angiogram done afterwards still showed some flow through the pseudoaneurysm (Fig. 3). So, another covered stent 34 mm overlapping the first one was placed. There was no flow noted in the pseudoaneurysm in completion imaging. (Fig. 4).

The procedure was uneventful and there were no immediate complications. The patient was discharged 2 days after the procedure. He was lost to follow-up, and returned to clinic at 6 months, where he was found to have good blood pressure control and continues to be on aspirin. Follow up echo showed the stents to be in a good position and confirmed its patency. He is gradually being weaned off the anti-hypertensive medications. We have planned an additional follow-up in 1 year with echo.

## Discussion

Blunt thoracic injury is one of the leading causes of death after motor vehicle accidents. Most of the patients having BTAI unfortunately die at the scene [10]. The treatment modality of aortic repair is often dictated by other associated injuries. Most of these patients have traditionally undergone a thoracotomy with or without an alternative perfusion pathway. They often require one lung ventilation if the associated injuries allow it. Historically, open repair has been associated with a high mortality rate reported at 28%, and spinal cord ischemia leading to paraplegia reported at 14% [11].

Thoracic endovascular repair for aortic injuries (TEVAR) is a relatively recent intervention that was approved by the FDA in 2005. This practice was very quickly adopted as an alternative method of management of BTAI. It involves placing an endovascular stent in the thoracic aorta at the location of injury from a remote access site under image guidance. Since its introduction there has been a consistent decline in the open surgery (OS).

Demetriades et al. compared TEVAR and OS after BTAI in adults [12]. In this prospective, observational multicenter study, 125 out of 193 patients examined had undergone TEVAR, with 68 of 193 having had OS. The patients in TEVAR group had significantly lower mortality (7.2% vs. 23.5 in OS group with OR of 8.42), blood transfusions (adjusted mean difference of 4.98 units) as well as procedure related paraplegia (0.8% vs. 2.9%) [12]. Stent related complications were reported in 20% of patients. The most common complication identified was endoleak, which was found in 18 cases (14.4%), 6 of which needed open repair. Other injuries included access vessel injuries (tear or thrombosis), occlusion of the left subclavian artery or left common carotid artery, stroke, paraplegia, and infection of the access site. Comparatively higher mortality and higher blood transfusion requirements were found in the OS group regardless of associated injuries [12].

In a landmark systematic review conducted by Murad et al., 139 studies were evaluated. Among the 7768 adult patients included, the mortality rate was lowest in those who underwent TEVAR (9%), followed by OS (19%) and highest in non-operative conservative treatment (46%) [13]. The risk of spinal cord ischemia, end stage renal disease, graft infection and systemic infections was higher with open repair compared to endovascular repair. Although, with endovascular repair an increased trend towards the need for a secondary procedure was seen [13].

The largest retrospective study in the pediatric population was done by Hasjim et al. in 2019 [14]. One-hundred and fifty nine patients with BTAI were identified over a period of 9 years (2007–2015). Of these, 124 (78.0%) underwent TEVAR and 35 (22.0%) underwent OS. Compared to patients undergoing OS, those with TEVAR had a shorter mean hospital length of stay (LOS) (16.4 vs. 21.4 days, *p* = 0.02) and ICU LOS (10.1 vs. 12.2 days, *p* = 0.01). There was no difference in the rates of mortality (5.5% vs. 8.6%, *p* = 0.56), or in in-hospital complications that included blood transfusions, acute kidney injury, pneumonia/ARDS, unplanned intubation, and cerebrovascular accident (*p* > 0.05) [14].

Our literature review yielded 23 other case reports of TEVAR for BTAI in the pediatric population. The majority of cases reported were for older children 8–17 years of age), with only one reported in a 16 month old child (See Table 1). The mortality rate was 13% (3/23), mostly attributed to associated injuries The mean follow up was 11 months (refer to Table 1).

**Table 1:** Descriptive summary of the literature review in pediatric population

Author	Report type	No. of pediatric patients	Age (years)	Stent	Complications	Treatment of complication	Survived?	Follow-up
Rousseau (1999) [15]	Case series	1	14	Not mentioned	None	–	Yes	11.6 months
Karmy-Jones (2002) [16]	Case series	3	12	PTFE/Palmaz	None	–	No	1 year
			12	AneuRX 22-mm cuff	None	–	Yes	
			14	Impra/Wallstent	None	–	No	
Wellons (2004) [17]	Case series	3	15	AneuRx 28- and 26-mm cuffs	None	–	No	Not mentioned
			16	Gore 23.5-mm cuff × 2	None	–	No	
			17	AneuRx 20-mm cuff	Endoleak	Repaired with additional AneuRx 20 mm cuff stent	Yes	
Milas (2006) [18]	Case series	2	16	23 mm × 2 endografts	None	–	Yes	13 months
			17	Medtronic 24 and 22 mm × 2 AneuRx	None	–	Yes	22 months
Saad (2006) [19]	Case report	1	12	Zenith 16 × 55 mm and 18 × 55 mm endografts	None	–	Yes	12 months
Azizzadeh (2009) [20]	Case report	1	14	Excluder (Gore)	None	–	Yes	Not mentioned
Gunabushanam (2009) [21]	Case report	1	11	AneuRx 16 mm	None	–	Yes	10 months
Goldstein (2012) [22]	Case series	4	14	2 Cheatham Platinum stent (20 × 39, 20 × 28 mm)	Stent fracture with no loss of integrity	–	Yes	24 months
			13	2 Cheatham Platinum stent (20 × 45, 20 × 34 mm)	None	–	Yes	
			14	Cheatham Platinum stent (18 × 39 mm)	Recurrent Aneurysm at 24 month follow up	Repaired with second Cheatham Platinum stent (18 × 39 mm)	Yes	
			11	Cheatham Platinum stent (16 × 39 mm)	None		Yes	
Hosn (2016) [23]	Case report	1	8	zenith alpha 18 mm x 105 mm	None	–	Yes	
Gombert (2016) [24]	Case report	1	15	stent prosthesis (ZTEG-2P- 24-115-PF)	None		Yes	6 months
Nicolas (2020) [25]	Case series	2	7	Cheatham Platinum (16 × 39 mm)	No			Not mentioned
			12	Talent Iliac limb stent graft (18 mm)	Endoleak	Repaired with 15 mm Palmaz Balloon mounted stent inflated to 18 mm	Yes	Not mentioned
Menini stahlschmidt (2010) [26]	Case report	1	11	–	–	–	Yes	6 months
Deeny S (2015) [27]	Case report	1	16 months	iCast stent grafts 16, 22 and 22 mm in overlapping fashion	No	–	Yes	6 months
Stringel (2018) [28]	Case Report	1	11	Cheetham platinum (14 × 45 mm)	Endoleak	Repaired with two Cheatham platinum stetns34 mm and 28 mm	Yes	20 months
Hasjim B.J (2019) [14]*	Retrospective review	124	16 (Median)	Multiple (summary in text)				4.5 years (mean)

*Case series with 9 patients out of which one patient fell in pediatric age group

The current guidelines in adult trauma by the society of vascular surgery (SVS) are based on the above mentioned review. The guideline suggests that endovascular repair of traumatic thoracic aortic injuries be performed preferentially over open surgical repair or nonoperative management, for grades II–IV. Non-operative management is recommended for Grade I [29]. This recommendation is based on very low quality evidence. There is unanimity of opinion that anatomic suitability is important for TEVAR but age should not be a factor in deciding the type of repair.

The use of TEVAR in pediatric BTAI is less established and is limited to case reports and case series. The anatomic challenge to the use of TEVAR is related to the small diameter of access vessels which may not accommodate the size of the delivery device. Currently no device has been approved by the FDA for traumatic aortic injury for the pediatric population, although off-label application of different endografts have been used.

According to SVC guidelines, TEVAR repair is best done within 24 h of injury if feasible, or at least prior to discharge (weak recommendation, low quality evidence) [29]. This was mainly based on adult literature. There is no such recommendation in children. In the case at hand, following a multidisciplinary discussion, we chose to delay the treatment for a weeks to allow for resolution and stabilization of associated injuries.

Given the rarity of the condition, there is very limited knowledge of the sequelae of endovascular management in this population. Long-term physiologic changes were depicted in a recent review of traumatic thoracic and abdominal injuries in otherwise healthy adults of age 45 ± 5.7 years [30]. These changes include increased aortic stiffness, blood pressure, cardiac mass, and aortic size after endovascular aortic repair in young patients suffering BTA [30]. The other concluded, however, that these effects might warrant close monitoring and management, but shouldn’t deter from choosing TEVAR over open repair. These effects might or might not be relevant in the younger pediatric patients. Some other potential long term concerns include stent occlusion, graft migration, pseudo-coarctation and durability of stent. Need for future open operative repair or upsizing of the stent also needs to be addressed. The incidence of these complications was not reported in pediatrics. The radiation burden and contrast related exposure is yet another concern in the younger population Therefore, long-term outcomes beyond 2 years have not been reported extensively. These are needed prior to establishing any recommendations in the pediatric age group.

## Conclusion

The management of BTAI in the pediatric population is an ongoing discussion. Although there have been a significant number of studies done in adults to evaluate the role of endovascular approach to repair the aortic injury, its use in the pediatric population has been limited to case reports and cases series. This case and others from literature highlight the short term safety and efficacy of TEVAR in the management of BTAI. The benefits and risks need to be evaluated on a case to case basis taking into consideration the site of injury, size of the patient, and associated injuries. Further studies are needed to decide on the long term effects of stenting in a young age, especially the durability of grafts, patency and their effect on aorta in the pediatric age group.
